# *Shewanella putrefaciens*: An Emerging Cause of Nosocomial Pneumonia

**DOI:** 10.1177/2324709618775441

**Published:** 2018-05-15

**Authors:** Saad Ullah, Hassan Mehmood, Nawja Pervin, Hassan Zeb, Khushbakht Ramsha Kamal, Sadaquat Liaqat

**Affiliations:** 1 Temple University/Conemaugh Memorial Medical Center, Johnstown, PA, USA; 2 Southern Illinois University, Springfield, IL, USA; 3 Fatima Jinnah Medical University, Lahore, Pakistan

**Keywords:** *Shewanella putrefaciens*, *Shewanella* algae, nosocomial pneumonia

## Abstract

Gram-negative infections are a rising concern faced by the medical community. Approximately 30% of nosocomial bloodstream infections in intensive care units in the United States are caused by these gram-negative species. Emergence of multidrug-resistant organisms further complicate this issue. In this article, we report a case of an 84-year-old Caucasian male who was diagnosed with *Shewanella* pneumonia treated with cefepime with minimal to no improvement in his symptoms. To the best of our knowledge, this is the third reported case of *Shewanella putrefaciens* nosocomial pneumonia and first case of bacteremia secondary to pneumonia by *Shewanella putrefaciens*.

## Introduction

Gram-negative infections are a rising concern faced by the medical community, particularly in the inpatient and intensive care settings. Emergence of multidrug-resistant organisms further complicates this issue and contributes to significant morbidity and mortality.^1^ Approximately 30% of nosocomial bloodstream infections in intensive care units in the United States are caused by these gram-negative species.^1^ *Shewanella putrefaciens* is an uncommon gram-negative bacilli, which commonly causes otitis media and soft tissue infections often after trauma or exposure to water sources.^2-4^ Rarely, it can lead to pneumonia and life-threatening bacteremia particularly in immunocompromised patients.^2-4^ In this article, we report a case of an 84-year-old Caucasian male who was diagnosed with *Shewanella* pneumonia treated with cefepime with minimal to no improvement in his symptoms. To the best of our knowledge, this is the third reported case of *S putrefaciens* nosocomial pneumonia and first case of bacteremia secondary to pneumonia by *S putrefaciens*.

## Case Presentation

**Figure 1.**
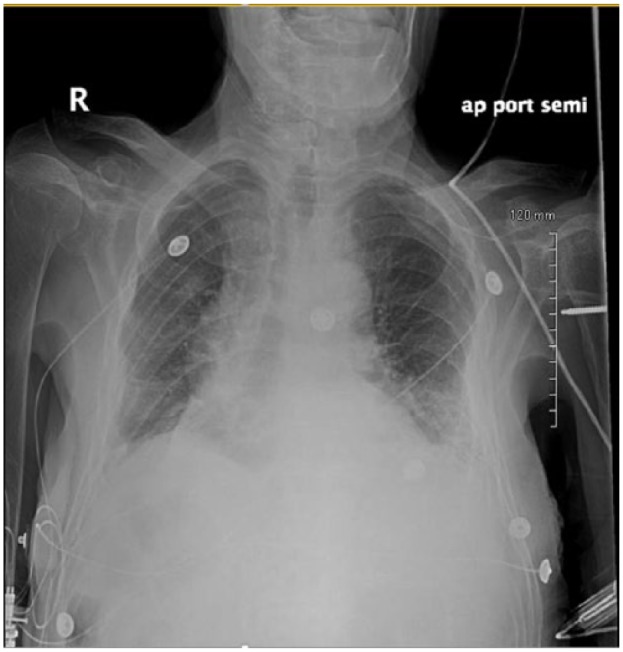
Chest X-ray showing bilateral bibasilar infiltrates with subtle bilateral pleural effusion more on the right side.

**Figure 2.**
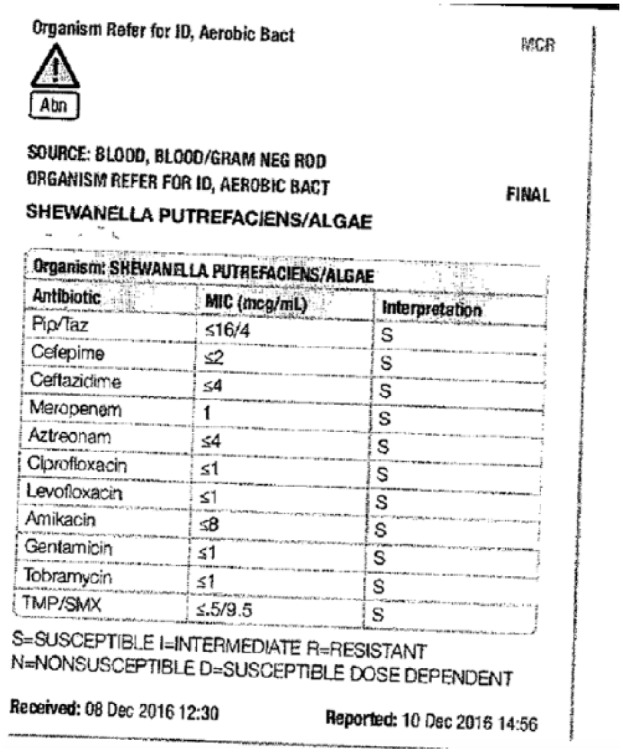
Blood culture sensitivity.

**Figure 3.**
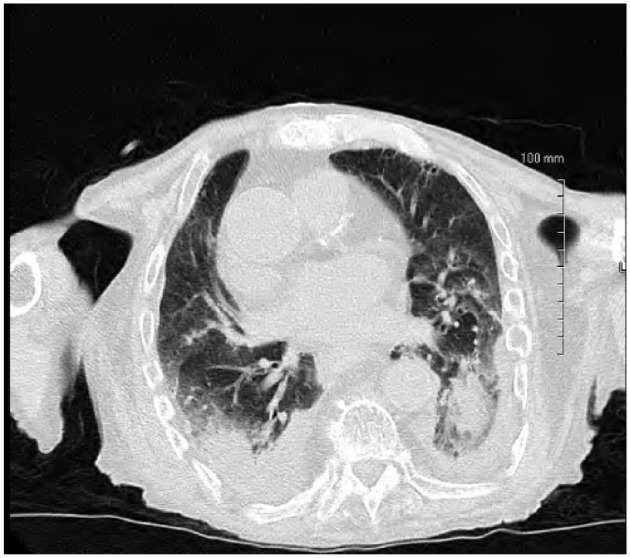
Computer tomography scan of the chest without contrast showed persistent bibasilar opacities with small to moderate right pleural effusion.

**Figure 4.**
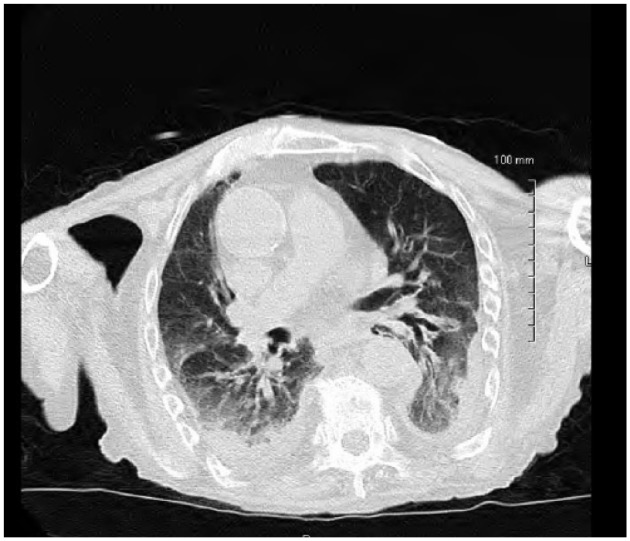
Computer tomography scan of the chest without contrast showed persistent bibasilar opacities with small left pleural effusion.

An 84-year-old Caucasian male with recent hospitalization for bleeding peptic ulcer disease and *Helicobacter pylori*–positive gastritis on amoxicillin, clarithromycin, and proton pump inhibitor presented to the hospital with fever, productive cough, and generalized fatigue for 5 days. He denied any sick contacts, recent travels, weight loss, night sweats, recent flu like illness, or exposure to marine sources, plant, or animal products. His other medical problems include chronic kidney disease, chronic obstructive pulmonary disease, stroke, prostate cancer treated with radiation therapy, and Paget’s disease. His vitals on admission include blood pressure of 110/75 mm Hg, temperature of 101.3°F, respiratory rate of 19 breaths per minute, pulse rate of 87 beats per minute, and pulse oximetry of 96% on 3 L nasal cannula. Physical examination was unremarkable other than decreased breath sounds more pronounced on the lower lobes with minimal expiratory rhonchi. Initial laboratory workup showed elevated white blood cell (WBC) count at 14.5 with neutrophilic predominance, stable hemoglobin of 13.5, and platelets of 488. Kidney function was normal with no electrolyte abnormalities noted on admission laboratory tests. Chest X-ray came back significant for bilateral lower lobe consolidation with left-sided pleural effusion (Figure 1). Two sets of blood, sputum, and urine cultures were sent, and he was started empirically on intravenous vancomycin and piperacillin/tazobactam for possible health care–acquired pneumonia. The patient remained febrile, and WBC count remained elevated with continuous production of thick, brownish colored sputum. Eventually, 2 sets of blood culture from admission and respiratory cultures came back significant for *S putrefaciens* susceptible to antibiotics given (as shown in Figure 2). Therefore, antimicrobials were tailored to culture and sensitivity and he was started on cefepime. WBC count trended down to 10 000 and subsequent blood cultures became negative. Despite negative blood cultures he remained febrile and had continuous thick and brownish secretions. Repeat sputum culture showed upper respiratory flora. Computer tomography scan of the chest without contrast was done, which showed persistent bibasilar opacities with small to moderate right pleural effusion and small left pleural effusion (Figures 3 and 4). Possible thoracentesis and bronchoscopy were planned. The patient responded to cefepime to some extent with negative blood cultures and trending down WBC count but at this point coinfection with other antimicrobial could not be ruled out as the patient was still spiking fever and thick brownish sputum. The patient’s prognosis considering his old age and multiple comorbid conditions was discussed with the family and they decided to pursue no further medical interventions including thoracentesis and bronchoscopy. Comfort care measures were initiated and he was transferred to palliative medicine service.

## Discussion

*Shewanella putrefaciens* is a rarely pathogenic, widely distributed, oxidase positive, nonfermenting, hydrogen sulfide forming gram-negative bacilli.^2-4^ These species are a part of microflora of marine environment and exposure to marine sources is considered an important risk factor for human diseases.^3,4^ It can also be found on other sources such as soil, dairy products, oils, poultry, and medical devices.^3-5^ Most cases are reported in warm climate areas and the bacteria is often isolated from cases of polymicrobial infections.^2-4^ Their virulence is well reported in humans although pathogenesis still remains uncertain.^3,4^ The *Shewanella* species of clinical significance include *S putrefaciens* and *S algae*.^3-5^ *Shewanella* species are fairly reported in literature to cause skin and soft tissue infections, ear infections, bacteremia, and hepatobiliary infections.^3-5^ Rarely they can cause lower respiratory tract infections, gastrointestinal infections, and hospital-acquired infections.^3,4^ These infections often result in setting of chronic diseases such as chronic liver disease, diabetes, chronic leg infections, infancy and immunocompromised hosts.^3,4^ The mechanisms by which these organisms are acquired in humans are by mucocutaneous abrasions or penetrating traumas with marine exposure, consumption of sea food or raw fish, or during aquatic recreational activities.^4^

Our patient presented with *Shewanella* bacteremia and pneumonia. The likely source and portal of entry cannot be identified. Although rare but there are reports in literature of respiratory colonization with possibility of infection of the lower tract.^2,5^ To the best of our knowledge, only 5 case reports have been reported in the literature of *S putrefaciens* pneumonia, with 2 of them classified as ventilator-associated pneumonia.^2,5^ Three of the reported 5 cases had identified exposure to river or sea water sources.^2,5^ Because of the recent hospitalization, it would be reasonable to categorize our patient as nosocomial pneumonia. According to our understanding, this is the third reported case of health care–associated pneumonia by *S putrefaciens* and first case that resulted in bacteremia from *S putrefaciens* pneumonia. *S putrefaciens* can be seen in sputum as a part of mixed flora or contamination, a situation that obscures the clinical significance of the organism’s presence.^5^ However, in our patient, positive sputum and blood cultures with clinical symptoms of pneumonia and supportive imaging leaves no doubt about the pathogenic character of this rare organism. As our patient had minimal to no improvement in his symptoms following the start of the usual susceptible regimen cefepime,^2,5^ what needs to be addressed at this time is the efficacy of different classes of antibiotics against this rarely pathogenic organism, widespread laboratory testing, and further studies on antibiotic susceptibility profiles.

## Conclusion

In conclusion, *S putrefaciens* is an uncommon gram-negative bacillus that commonly causes otitis media and soft tissue infections often after trauma or exposure to water sources. Only 2 cases of *S putrefaciens* causing pneumonia have been reported so far. We recommend raising awareness in the health care profession about *S putrefaciens*. Physicians should keep in mind the efficacy of different classes of antibiotics against this rarely pathogenic organism. It is too early to comment on the best choice of antibiotic as limited data are available due to the rarity of this organism. *S putrefaciens* responded well to cefepime in most of the case reports.

## References

[1] Peleg AY Hooper DC. Hospital-acquired infections due to gram-negative bacteria. N Engl J Med. 2010;362:1804-1813. 20463340 10.1056/NEJMra0904124 PMC3107499

[2] Patel R Abraham A Thomas J Zhi W Ahmed S Verley J. A rare case of pneumonia caused by *Shewanella putrefaciens*. Case Rep Med. 2012;2012:597301. 10.1155/2012/597301 PMC3485528 23133456

[3] Vignier N Barreau M Olive C et al Human infection with *Shewanella putrefaciens* and *S algae*: report of 16 cases in Martinique and review of the literature. Am J Trop Med Hyg. 2013;89:151-156. 23690548 10.4269/ajtmh.13-0055 PMC3748472

[4] Janda JM. *Shewanella*: a marine pathogen as an emerging cause of human disease. Clin Microbiol Newsl. 2014;36:25-29.

[5] Tucker C Baroso G Tan P. Ventilator-associated pneumonia due to *Shewanella putrefaciens*. Am J Health Syst Pharm. 2010;67:1007-1009. 20516471 10.2146/ajhp090344

